# Spectroscopic Characterization and Nanosafety of Ag-Modified Antibacterial Leather and Leatherette

**DOI:** 10.3390/nano7080203

**Published:** 2017-07-29

**Authors:** Maria Chiara Sportelli, Rosaria Anna Picca, Federica Paladini, Annarosa Mangone, Lorena Carla Giannossa, Cinzia Di Franco, Anna Lucia Gallo, Antonio Valentini, Alessandro Sannino, Mauro Pollini, Nicola Cioffi

**Affiliations:** 1 Dipartimento di Chimica, Università degli Studi di Bari “Aldo Moro”, Via Orabona 4, 70126 Bari, Italy; maria.sportelli@uniba.it (M.C.S.); annarosa.mangone@uniba.it (A.M.); lorenacarla.giannossa@uniba.it (L.C.G.); 2 CNR-IFN U.O.S. Bari, Via Amendola 173, 70126 Bari, Italy; cinzia.difranco@uniba.it; 3 Dipartimento di Ingegneria dell’Innovazione, Università del Salento, Via per Monteroni, 73100 Lecce, Italy; federica.paladini@unisalento.it (F.P.); annalucia.gallo@unisalento.it (A.L.G.); alessandro.sannino@unisalento.it (A.S.); mauro.pollini@unisalento.it (M.P.); 4 Dipartimento Interateneo di Fisica, Università degli Studi di Bari “Aldo Moro”, Via Amendola 173, 70126 Bari, Italy; antonio.valentini@uniba.it

**Keywords:** silver nanoparticle, antibacterial, nanosafety, X-ray photoelectron spectroscopy (XPS), single particle inductively coupled plasma mass spectrometry (ICP-MS), nanoparticle release

## Abstract

The development of antibacterial coatings is of great interest from both industry and the consumer’s point of view. In this study, we characterized tanned leather and polyurethane leatherette, typically employed in the automotive and footwear industries, which were modified by photo-deposition of antibacterial silver nanoparticles (AgNPs). Material surface chemical composition was investigated in detail by X-ray photoelectron spectroscopy (XPS). The material’s antibacterial capability was checked against *Escherichia coli* and *Staphylococcus aureus*, as representative microorganisms in cross transmissions. Due to the presence of silver in a nanostructured form, nanosafety issues were considered, as well. Ionic release in contact media, as well as whole nanoparticle release from treated materials, were quantitatively evaluated, thus providing specific information on potential product nanotoxicity, which was further investigated through cytocompatibility MTT (3-(4,5-dimethylthiazol-2-yl)-2,5-diphenyltetrazolium bromide) assays, also after surface abrasion of the materials. The proved negligible nanoparticle release, as well as the controlled release of antibacterial ions, shed light on the materials’ potentialities, in terms of both high activity and safety.

## 1. Introduction

Consumers attention towards hygiene-safe leather and leatherette products has been growing over the years, because of the spreading of drug-resistant infections [1,2]. It has then been necessary to tackle the antimicrobial resistance phenomenon in several public (e.g., public transport) and private contexts (e.g., footwear and clothing). Such surfaces, apparently inert, are frequently reservoirs for many infections. Most common pathogens may, in fact, survive on these substrates for months, becoming a continuous source of cross-transmission diseases [3]. The persistence of pathogens on inanimate surfaces in public and domestic places greatly depends on the type of microorganism; for example, some bacteria die within a few minutes during drying, other bacteria, such as *Escherichia coli*, can survive on dry surfaces for months [4,5]. Leather, namely tanned animal skin composed of a complex collagen network, can provide ideal conditions such as moisture, temperature, oxygen, and nutrient required levels for the rapid growth of bacteria and fungi [6]. Synthetic leather simulates the natural leather microstructure, thanks to the presence of superfine polyurethane (PU) fibers resembling the fineness and structure of animal skin fibrils [7]. Like genuine leather, however, PU leatherette is also susceptible to microbial contamination. First, the polyester segments of PU macromolecules can serve as carbon sources for microbial growth [1]. Second, extra components in the PU coating, such as plasticizers, lignocelluloses, stabilizers and colorants, can also be partially responsible for microbial attack [8]. Once contaminated, both natural and PU leathers will become degraded, discolored, and odorous; such problems will shorten the product’s lifespan and quality [9]. In addition, microbial contamination results in increased infection danger and the harmful influence of toxins, which severely threaten human health [10]. For example, in footwear, surface colonization and microorganisms’ growth could cause skin infections that can be quite lengthy, difficult to eradicate, and damaging [11]. In public transport, the high number of passengers in trains, buses, planes etc., cannot assure a proper degree of hygiene, thus causing allergies, dermatitis or skin infections [12,13,14]. To overcome this problem, many antimicrobial agents are employed industrially to manufacture leather and leatherette coatings with antimicrobial function. Especially in the case of natural leather, post-tanning and finishing operations, designed to protect the leather surface from scratches and abrasions, and to make it pleasing to the eye and to the touch, are often performed. Generally, they consist of the deposition of a thin polymer layer; in most cases, PU is used, being a good compromise among resistance, cheapness, transparency, and color retention [15]. Many manufacturing industries have exploited this post-tanning treatment to confer antimicrobial properties to both leather and leatherette, by simply mixing the bioactive agent into the polymeric dispersion. Unfortunately, many of the chemicals and strategies used to impart antimicrobial property to these materials are not green, usually toxic to humans, and lead to the proliferation of antibiotic-resistant bacteria [16]. Thus, the development of eco-friendly and effective antimicrobial coatings for natural and manufactured leather products would provide benefits to both industry and consumers. Having a strong antimicrobial activity, silver nanoparticles (AgNPs) have been attracting great interest [17]. Many examples dealing with silver-based antimicrobial coatings for leather and leatherette materials are reported in the literature. The most common method used to apply nanostructured coatings to substrates is pad-dry cure [18]. This way, AgNP suspensions with the desired concentration can be easily applied to almost any textile surface [19]. Sometimes, after application of Ag-based colloids and proper drying, the treated surface is ironed, in order to form a thin film containing AgNPs on it [6]. Simple spreading of AgNPs on surfaces is generally avoided: due to the presence of the polymeric protecting layer, a proper NP adhesion on the substrate is not guaranteed, causing potential nanotoxicological issues related to whole nanoparticle release in contact media. This drawback was successfully overcome by Maestre-López and co-workers, immobilizing AgNPs in a SiO_2_ matrix [20]. Recently, green-synthesized AgNPs were applied to a leather surface by simple immersion of specimens in diluted colloidal suspensions under continuous sonication [21]. The same methods here listed were applied to other bioactive NPs, like Au [22] and TiO_2_ [1] ones. Analogously, antimicrobial compounds like chitosan (CS) [23], plant extracts [24], and essential oils [15], were mixed with the protective PU layer to produce safe antimicrobial leather and leatherette. In this study, a patented technology developed at the University of Salento (Italy) was adopted to deposit silver coatings on both natural leather and leatherette [25]. This technology is based on the photoreduction of a silver solution directly onto the desired substrate, which provides a uniform cluster distribution and a good coverage. An impressive antimicrobial capability against fungi and Gram-negative and Gram-positive bacteria [26] was already demonstrated in our previous work, the latter being retained also after abrasion tests, suggesting that silver-treated materials preserve their efficacy in any condition of use [9,12]. Here, we mainly focus on the detailed surface characterization of modified leather/leatherette samples by means of X-ray photoelectron spectroscopy (XPS) as well as on the assessment of silver release (both in particulate and ionic form) in real-life conditions. As the modification process is already patented [25], here we aimed at fully characterizing these innovative materials from the morphological, spectroscopic, and biological point of view. We combined for the first time results arising from inductively coupled plasma mass spectrometry (ICP-MS), operating both in classical and single-particle (SP-ICP-MS) modes. Such results were confirmed by transmission electron microscopy (TEM) measurements on the same contact solutions. In fact, AgNP-containing products may lead to (nano-) particle release; this phenomenon could raise concerns for the environment and humans. For this purpose, biological characterizations were performed to assess the antibacterial capability of silver treated substrates and their cytocompatibility. All the biological tests were performed before and after Taber abrasion tests, aiming at the evaluation of the durability of the antibacterial properties and the potential cytotoxicity associated with the release of silver from the substrates under any condition of use. The antibacterial properties were evaluated by agar diffusion tests on *Escherichia coli* and *Staphylococcus aureus*, according to the Standard “SNV 195920-1992”, and by bacterial enumeration tests through the serial dilution method. MTT assays were performed on murine fibroblasts through the indirect method. Hence, the nanosafety of Ag-modified leather products was here assessed, paving the way for their harmless application in real-life products, both in private and social contexts.

## 2. Results and Discussion

### 2.1. Surface Modification of Leather and Leatherette

**Figure 1:**
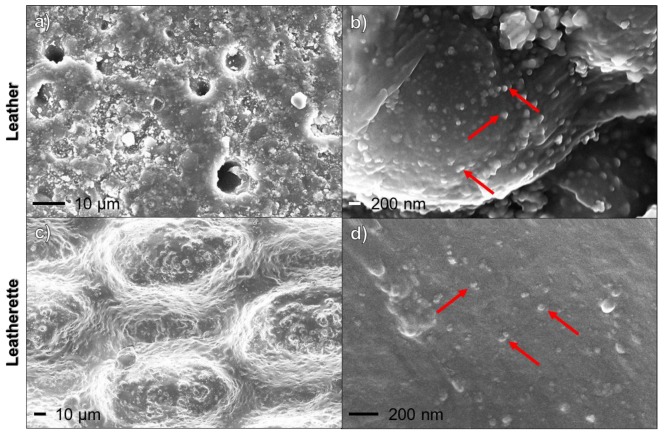
Scanning electron microscopy (SEM) morphological characterization of Ag-modified leather ((**a**,**b**) panels, different magnifications) and leatherette ((**c**,**d**) panels, different magnifications) samples. Arrows highlight inorganic clusters on samples surfaces.

Leather and leatherette substrates were modified with AgNPs by *in-situ* photoreduction of silver nitrate, as described in the following section. Pristine and treated specimens were subjected to SEM characterizations, in order to verify silver particles distribution onto the sample surfaces (Figure 1). Mostly large, inorganic spheroidal clusters were detected. In fact, contrast in SEM microscopy is enhanced by the atomic number (*Z*), leading to a brighter image for elements with higher *Z* values (e.g., Ag). In Figure 1, small clusters, which appear brighter, can be related to inorganic matter, with a high atomic number. On the contrary, organic polymer (i.e., polyurethane) gives a low-contrast background.

### 2.2. Surface Characterizations

Both treated and pristine samples were analyzed by XPS. Table 1 reports surface atomic percentages (At%) of leather samples. It is worth mentioning that leathers coated with a polyurethane layer were employed in this work. As a result, XPS revealed the presence of typical elements of PU-leather substrates (C, O, N) as well as trace elements (halogens), which were considered as contaminants. The occurrence of a relatively high silicon content was not surprising, considering that the analyzed leathers had undergone industrial finishing and anti-scratch procedures. Silver was easily detectable after photo-reduction treatments, confirming that such a method can be considered reliable and efficient on almost every fabric substrate.

**Figure 2:**
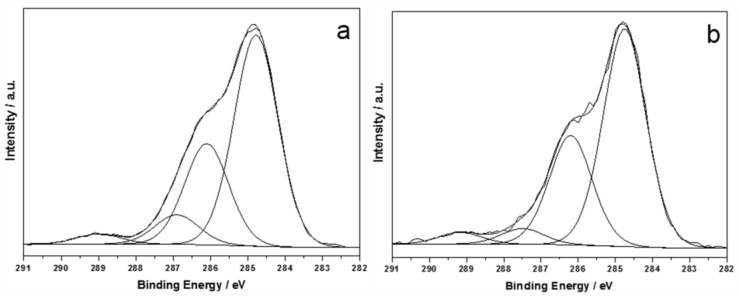
C1s X-ray photoelectron high-resolution region (XP-HRR) of pristine leather (**a**) and Ag-treated leather (**b**).

Surface chemical composition was fairly uniform, as observable from the low values of standard deviations reported in Table 1. These results are indicative of the presence of a good distribution of antibacterial AgNPs on treated leather. High-resolution regions of main elements were subjected to detailed curve fit procedures, in order to evaluate changes in chemical speciation after the antibacterial treatment. C1s region was evaluated first. A comparison between spectra relevant to pristine and treated samples is reported in Figure 2.

Signal component attributions, positions, and relative abundances are listed in Table 2.

**Figure 3:**
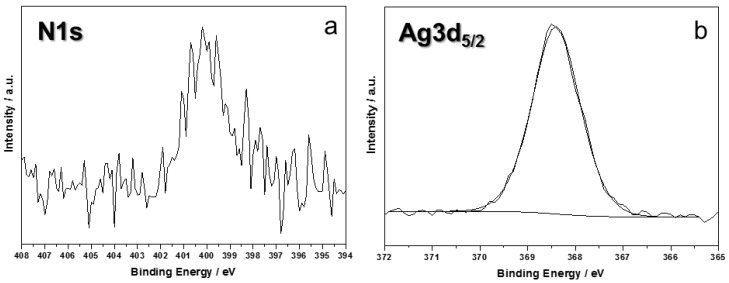
N1s (**a**) and Ag3d_5/2_ (**b**) XP-HRR of treated leather.

It is worth noting how all chemical environments associated with carbon were not significantly influenced by the presence of Ag clusters. Moreover, signal components are in agreement with what is expected for PU-coated materials [27]. N1s region was extremely important, being characterized by different binding energy (BE) values for different types of organic nitrogen (e.g., C≡N, C=N, N–C=O, typical of polyurethane and proteins), which fall at 399 and 402 eV, and inorganic nitrogen (e.g., derived from AgNO_3_ silver salt) that is observable at BE = 407 ± 1 eV. XPS analyses allowed us to verify the absence of silver precursor on treated samples: no signal components were detectable above 403 eV. These findings proved that the patented process here described leads to a complete reduction of silver salts into nanoclusters, deposited on leather. A typical N1s spectrum for modified leather is reported in Figure 3a.

Deconvolution of Ag3d_5/2_ signal returned a single component, centered at 368.4 ± 0.2 eV (Figure 3b). This position is compatible with Ag^0^, although the presence of Ag^+^ cannot be completely averted, because its BE is very similar to the one associated with elemental silver [28]. Moreover, due to the very low silver At%, study of AgM_45_N_45_N_45_ main Auger transition was not possible, this component suffering a very low signal-to-noise ratio.

Surface elemental composition of the leatherette samples (BENOVA^®^ type) is summarized in Table 3. Elements typical of polyurethane-based materials were identified, as well as significant amounts of silicon. Surface percentages showed, also on these samples, excellent point-to-point reproducibility.

**Figure 4:**
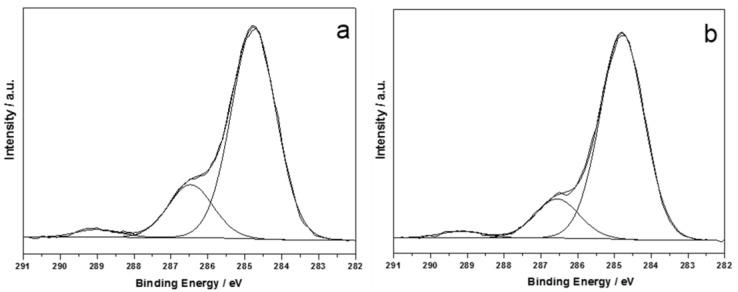
C1s XP-HRR of pristine leatherette (**a**) and treated leatherette (**b**).

As expected, treated leatherette exhibited the presence of silver, as well. Figure 4 shows typical C1s spectra, relevant to pristine and treated PU leatherette.

C1s high-resolution regions were made up of three components, compatible with those expected for polyurethane materials, whose position and relative abundance is similar for pristine and treated samples (Table 4).

**Figure 5:**
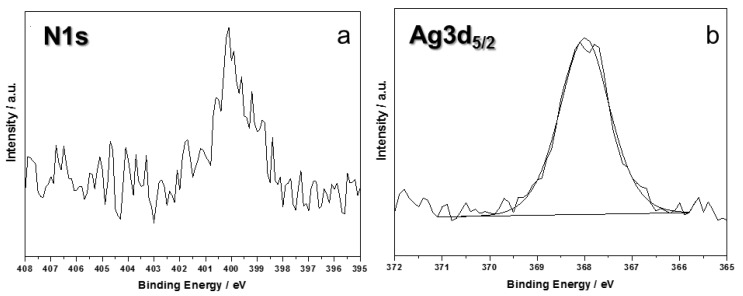
N1s (**a**) and Ag3d_5/2_ (**b**) XP-HRR of treated leatherette.

Comparing the XPS data on natural leather and leatherette, it was evident how the organic fraction of both industrial substrates did not undergo significant alterations when subjected to Ag nanoclusters deposition. Analysis of N1s region (Figure 5a), revealed the presence of one component at BE ~400 eV for both the pristine and treated leatherette. Presence of unreacted silver nitrate precursor was excluded, analogously to natural leather samples. Ag3d_5/2_ region (Figure 5b) was made up of a single component at 368.0 ± 0.2 eV, compatible with nano Ag^0^. Similar to the case of natural leathers, Auger AgM_45_N_45_N_45_ region did not allow any further data treatment and curve-fitting procedures, because of its low intensity.

### 2.3. Evaluation of Ionic Release

**Figure 6:**
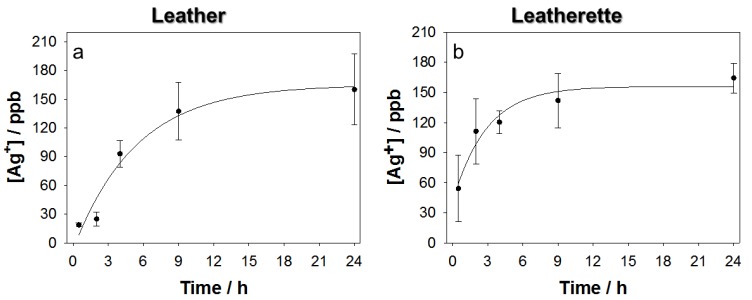
Silver ion release kinetics from Ag-treated leather (**a**) and leatherette (**b**).

Natural and PU leather samples, treated with 4% *w*/*w* silver, were first placed in contact with 2% *w*/*w* HNO_3_ for 24 h, in order to roughly estimate the maximum amount of Ag^+^ releasable from each specimen. It is in fact known that nitric acid is a strong oxidizing agent, used to fully dissolve metal species and perform trace metal analysis. All samples showed a total release in the range of tens–hundreds of ppb, well below the values found for other PU-based substrates, like Ag-modified filters and padding foams [29]. In this medium, both samples exhibited a relatively low release of silver ions, probably due to the intrinsic nature of the modified substrates, which are characterized by a higher retention of silver nanophases. Moreover, the limited porosity of the materials may hinder ion diffusion from the innermost layers. Untreated specimens were used as control samples, and showed a negligible silver ion release when immersed in PBS for 24 h. In fact, the average release measured for untreated leather and leatherette was equal to 3.8 ± 0.7 ppb and 1.8 ± 0.4 ppb, respectively. On the contrary, silver-modified materials exerted a significant and modulated ionic release over time. Figure 6 shows typical trends of ionic release kinetics obtained for both leather and leatherette.

The maximum concentration registered on triplicate analyses resulted below 200 ppb, a value that is much lower than the one considered “critical” in terms of human toxicity for Ag^+^ ions (~1 ppm). Although low, such ion release is capable of exerting a good antimicrobial activity. Moreover, unlike other polyurethane-based materials [29], which provided an immediate and higher ionic release, in the present case, a time trend is observable for [Ag^+^], following pseudo-first-order kinetics. It is worth noting that, starting from a constant precursor concentration, the photo-deposition process results in different ion release extent and kinetics, being a function of the deposition material, as outlined by different release features observed for cotton fabrics [30], PU foams [29] etc.. Release from leatherette appears faster: this is probably due to a higher roughness of the leatherette surface with respect to leather.

### 2.4. Assessment of Entire Nanoparticle Release

Single particle inductively coupled plasma-mass spectrometry (SP-ICP-MS) has proven to be a powerful technique for the detection and characterization of metal-containing nanomaterial dispersions [31]. Combining the high-throughput of a single-particle counting technique and the elemental specificity of ICP-MS, SP-ICP-MS is capable of providing information about NP size distribution, and concentration with minimal sample pre-treatment [32]. The principle of SP-ICP-MS relies on a continuous reading of a single mass, with a short dwell time. In particular, the output signal resulting from measuring dissolved ions is quite different from the signal derived from the detection of single particles present in solution. In fact, in the case of ionic species (such as Ag^+^), a steady state signal can be acquired over time (expressed in terms of number of readings), whose average intensity is proportional to ionic concentration. On the other hand, NPs present in solution produce in the plasma a cloud of ions for each particle, detected as a discrete peak for each particle. In this case, the number of pulses above the background represents the number of the individual NPs detected.

**Figure 7:**
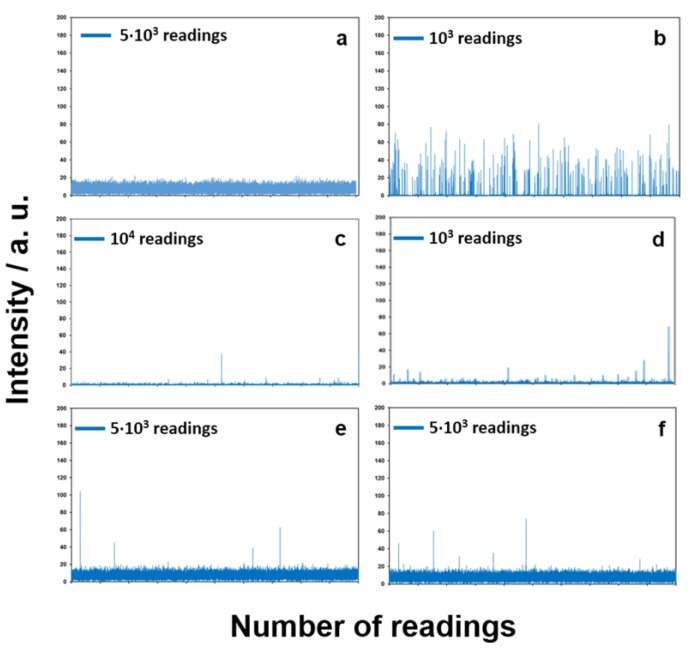
Single particle inductively coupled plasma-mass spectrometry (SP-ICP-MS) spectra of control 1 μg/L Ag^+^ solution (**a**); control AgNP suspension (**b**); solution in contact with untreated leather (**c**); solution in contact with untreated leatherette (**d**); solution in contact with treated leather (**e**); solution in contact with treated leatherette (**f**). *Y*-axis goes from 0 to 200 a.u. with 20 a.u. increment, in all panels. Scale bars indicate number of data points along *X*-axis.

As a result, the SP-ICP-MS technique enables the simultaneous determination of two independent parameters, e.g., (i) the concentration of dissolved metal ions (which gives a continuous—and apparently noisy—background), and (ii) the concentration of entire nanoparticles (which is related to the frequency of “pulses”/spikes superimposing on the background), along with their size (which is related to the pulse intensity). All of these parameters can be obtained at very low concentrations. When analyzed by SP-ICP-MS, the two control samples (i.e., AgNP colloid prepared by chemical reduction, and AgNO_3_ solution) gave very different results. In fact, with this analysis mode, acquiring the ^107^Ag^+^ signal over time, a continuous background was obtained in the presence of silver ions (Figure 7a), while discontinuous/pulsed spikes were visible for colloidal suspensions (Figure 7b). In the latter case, each pulse corresponded to the atomization of a single particle. Furthermore, the intensity of each pulsed signal was directly related to NP size, while pulse frequency was associated with particle concentration [10]. Analysis of real samples, in which both NPs and dissolved ions are present, understandably shows the superimposition of pulses (due to particles), on a continuous background (due to the presence of ions, exerting the antibacterial activity). SP-ICP-MS data obtained from solutions let in contact with leather and leatherette samples depicted interesting and reassuring releases in terms of toxicological liability. Figure 7 shows typical signals for all the samples here investigated, namely standard/control samples, e.g., (a) 1 μg/L AgNO_3_ solution (resembling the sole ionic release), (b) AgNPs colloidal suspension (mimicking only NP release), as well as contact solutions for pristine (c,d) and AgNP-modified (e,f) leather or leatherette materials. Release spectra for treated leather and leatherette (panels e and f) depicted a continuous background, attributable to Ag^+^ ions, with an average intensity of 20 a.u. (arbitrary units). A few individual pulses were easily distinguishable as well, with variable intensities. These pulsed signals were attributable to the presence of nano/micro particles in the contact solution. Pulse frequencies did not differ significantly between pristine and blank samples. Indeed, when injecting the control AgNP suspension (panel b), a very dense series of pulsed and discrete signals was obtained. Such pulses laid on a very low background, due to the almost total absence of dissolved ions in solution. Different pulse intensity was due to different NP sizes in their suspension; rarely, are intense pulses associated with the simultaneous atomization of two (or more) particles. The analysis of a silver salt solution produced a continuous background signal (panel a), whose average intensity was proportional to [Ag^+^] in solution (1 μg/L). Comparing all spectra, we could conclude that Ag-treated materials can exert a good ionic release, with limited toxicological risks associated with the eventual loss of entire NPs. In fact, only a few events attributable to the release of whole micro- and nano-particles were observed. The presence of a certain ionic background registered for treated samples was in agreement with ICP-MS data on ionic release kinetics. Finally, it is worth noting that, on real samples, pulse intensities are generally higher than the ones reported for the Ag colloid. Hence, it could be inferred that the few released silver clusters certainly have a mean diameter greater than 50 nm. In fact, intensities obtained for real samples ranged between 40 a.u. and 100 a.u., while the AgNP reference colloid, containing 50-nm AgNPs, generated pulses of about 40 a.u..

**Figure 8:**
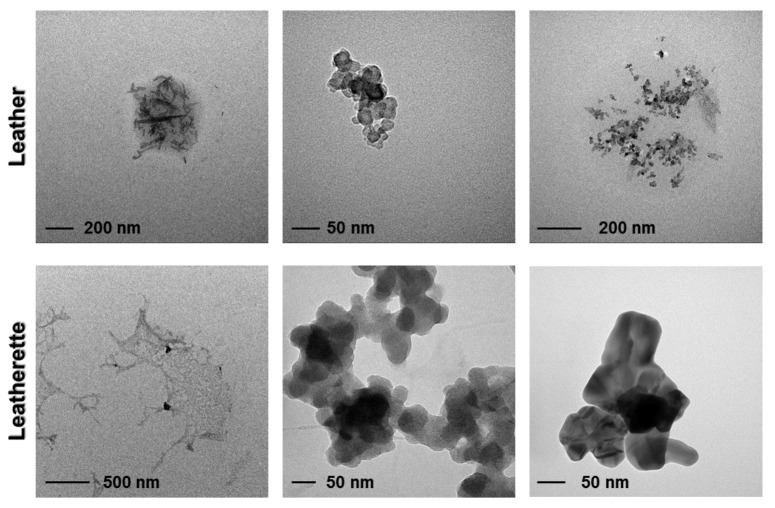
Transmission electron microscopy (TEM) micrographs of solutions in contact with treated leather (upper panels) and leatherette (lower panels). Most of the analyzed fields showed no inorganic particle at all. The few fields containing any measurable inorganic particle/aggregate are shown herein.

This evidence was further encouraging, since a particle diameter of 50 nm is usually considered the alert threshold for possible occurrence of toxicological risks [33]. SP-ICP-MS indirect results were further validated by performing TEM characterizations on aliquots of the same contact solutions (refer to Section 3 for further experimental details). This enabled a direct evaluation of the eventual presence on nanostructures coming from treated materials in the contact solution. Figure 8 shows some micrographs obtained for treated leather and leatherette specimens, for explanatory purposes. These images represent a selection among numerous ones, acquired on different grids, exploiting microscope tracking mode (analyzed area ~5 mm^2^). Most of the examined fields showed the total absence of any detectable inorganic particle. Only in rare cases, were micro- or nanoparticles observed. These clusters appeared irregular in both shape and dimension, and could not be associated with individual AgNPs released from treated materials. No substantial differences in structures contrast were observed between samples, indicating how the observed particles could be predominantly ascribed to occasional environmental contamination (i.e., dust particulate). Moreover, in both cases, clusters of organic/inorganic mixed composition were visible, in which a low-contrast (organic) background surrounded various elongate/fibrous (inorganic) structures. Such similarity suggests that released particles could arise from portions of the samples released as composite clusters, including leather/leatherette tanning and finishing residuals, rather than being correlated to the presence of bare, small sized—and more reactive—AgNPs.

### 2.5. Biological Characterizations

#### 2.5.1. Antibacterial Tests

**Figure 9:**
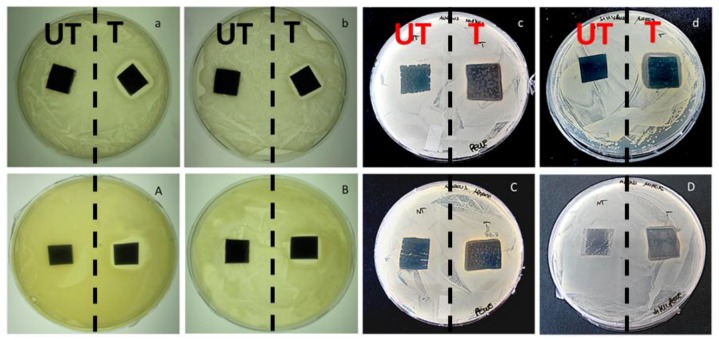
Agar diffusion tests on *E. coli* before (a,b) and after (A,B) surface abrasion on pristine and Ag-treated leather (**a**,**A**), pristine and Ag-treated leatherette (**b**,**B**). Agar diffusion tests on *S. aureus* before (**c**,**d**) and after (**C**,**D**) surface abrasion on pristine and silver-treated leather (**c**,**C**), pristine and silver-treated leatherette (**d**,**D**). Dashed lines separate untreated samples (labeled as UT) from Ag-modified ones (labeled as T), present respectively on the left and on the right of each Petri dish.

The antibacterial potentialities of the materials herein investigated were checked in standard microbiological tests. Results of Agar diffusion tests are reported in Figure 9. Although a very low amount of silver was used for this characterization, a well-defined inhibition area to *E. coli* and *S. aureus* growth was visible around silver-treated leather and leatherette before (Figure 9a–d) and after (Figure 9A–D) the surface abrasion. The Antibacterial Efficacy percentage (ABE %) of modified leather and leatherette was also quantified through bacterial enumeration tests performed before and after surface abrasion. Representative pictures of agar plates adopted for bacterial counts are reported in Figures S2 and S3 for all the samples. Before abrasion, ABE % on *E. coli* resulted in 98.9 ± 0.7% and 99.1 ± 0.7% on leather and leatherette respectively, and 96.1 ± 0.7% and 98.9 ± 0.8% respectively on leather and leatherette after the abrasion test. ABE % on *S. aureus* was found to be 98.1 ± 0.6% and 98.9 ± 0.7% on leather and leatherette respectively before the abrasion test, and 98.9 ± 0.8% on leather and 97.1 ± 0.9% on leatherette after abrasion. These results confirm a good efficacy of the silver-treated materials, along with an excellent durability of the silver coatings, which demonstrated good antibacterial efficacy even after aging of the samples.

#### 2.5.2. Biocompatibility of Leather and Leatherette

**Figure 10:**
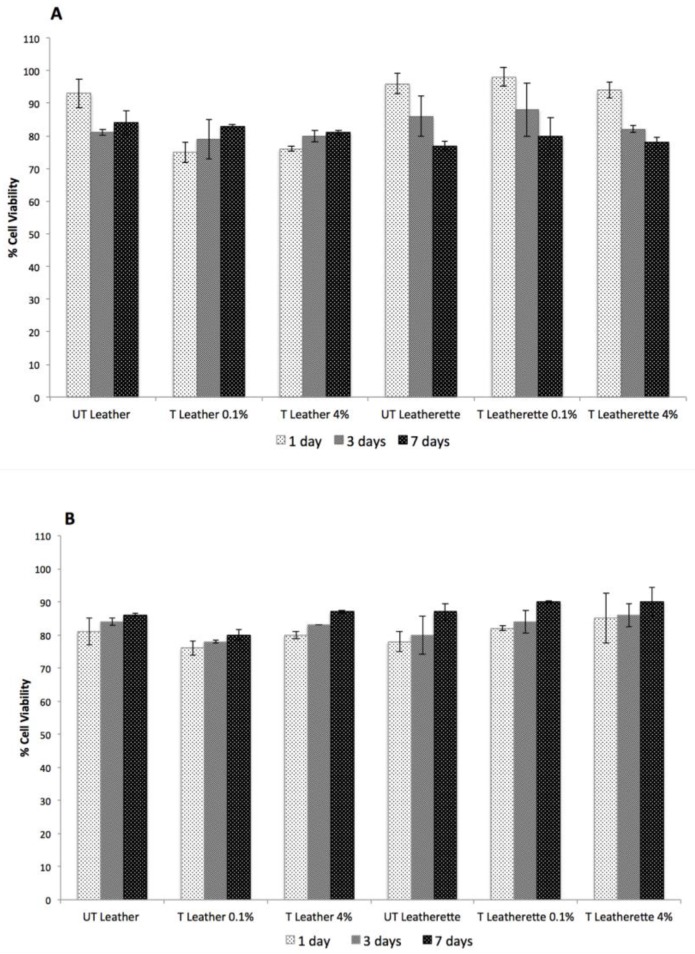
MTT assays at 1, 3 and 7 days before (**A**) and after (**B**) the abrasion test. UT = untreated, T = treated; 0.1% and 4% represent the weight percentages adopted for the treatment of the samples.

In vitro biocompatibility of Ag-treated samples was determined on 3T3 cells according to the indirect extraction method, in order to assess the safety of the materials related to the release of silver from the coatings. Both the highest and the lowest Ag precursor concentrations were tested, namely 0.1 and 4% *w*/*w*, and this was carried out also after the Taber test. The viability of murine fibroblasts 3T3 cultured in the presence of extracts from the silver treated materials was assessed by MTT assay at 1, 3, and 7 days and the results are reported in Figure 10 before (Figure 10A) and after (Figure 10B) abrasion. Extracts from Ag-treated samples did not affect significantly (*p* > 0.05) cell viability with respect to control samples at any time point, thus indicating that the silver treatment does not determine adverse effects for health associated to silver release. Moreover, statistically significant differences were not observed in cell proliferation also after abrasion, further confirming the nanosafety of the materials.

## 3. Materials and Methods

### 3.1. Surface Modification of Leather and Leatherette

Natural tanned leather and PU leatherette (BENOVA^®^ type) were kindly provided by Tecnofibre Srl (Avellino, Italy). 50 × 50 cm^2^ squares were modified with AgNPs by adopting a patented protocol [25] based on a photo-assisted reduction process of a silver nitrate (>99%, Alfa Aesar, Milan, Italy) solution (4% *w*/*w*) spray-deposited (100 g/m^2^) onto sample surfaces. Silver percentage was chosen as the highest possible silver concentration which can be deposited on a real leather/leatherette substrate. Silver solution was prepared in water and methanol (99.9%, Sigma Aldrich, Milan, Italy), the latter acting as both solvent and reducing agent. Wet substrates were finally exposed to an ultraviolet source (Jelosil HG 1000, λ = 365 nm, working distance = 50 cm) for 10 min each. Through this step, silver precursor (Ag^+^) was photo-reduced to Ag^0^. The morphology of deposited clusters was evaluated by a field emission scanning electron microscope (FE-SEM), model Σigma Zeiss (Jena, Germany). Prior to analysis, samples were coated with a 5-nm Pd layer, to avoid surface charging. They were examined at a 10-kV acceleration voltage, using a 30 μm aperture and in-lens detector.

### 3.2. XPS Surface Characterization

Both pristine and Ag-treated samples were analyzed by XPS, in order to assess their surface chemical composition and silver chemical state. Measurements were performed on a Thermo Theta Probe VG Scientific, equipped with a monochromatized AlKα X-ray source, with a 300-μm spot size, in constant analyzer energy (CAE) mode. Samples were attached onto the sample holder by means of a double-sided conductive copper tape. Due to their insulating nature, charge compensation was applied during analysis, using a 10^−8^ mbar Argon flow as ionization gas. Exposure time to X-ray radiation was optimized as a good compromise between signal-to-noise ratio and minimum sample damaging. The latter was evaluated comparing proper reference signals (C1s for pristine samples, C1s and Ag3d for treated ones) before and after a whole spectra acquisition. Spectra processing was performed by the Thermo Avantage^®^ software (v. 5.937, 2014, Thermo Fisher Scientific, East Grinstead, UK): XPS surface elemental compositions were obtained after *Shirley* background removal, as mean values over three different replicates.

### 3.3. Evaluation of Silver Ion Release

Silver ion (Ag^+^) release was quantified by ICP-MS on 100 mg/cm^2^ and 70 mg/cm^2^ leather and leatherette samples, respectively. Ion release kinetics were evaluated by soaking samples in a phosphate buffer saline (PBS) solution, resembling physiological conditions. High purity salts (TraceSelect^®^, Sigma, Italy), were used to prepare PBS, mixing phosphate buffer (pH = 6.8, ionic strength I = 0.1) with 0.85% *w*/*w* NaCl, in 1:1 ratio. Samples were let in contact with 6 mL of PBS solution in screw-cap 25-mL glass vials. At defined times (30 min, 2 h, 4 h, 9 h, 24 h), the liquid medium was sampled and analyzed by ICP-MS. All experimental data were averaged on at least three replicates. Concentration of silver ions in each contact solution was evaluated by a NexION 300-X Perkin-Elmer ICP-MS spectrometer (Table S1), equipped with a collision cell, monitoring isotopes ^107^Ag and ^109^Ag. Indium was used as internal standard, with a final concentration of 25 ppb. The spectrometer was calibrated by Ag^+^ solutions, obtained by adding known amounts of TraceSelect^®^ AgNO_3_ in 6 mL of PBS, and reaching a final volume of 50 mL with 2% *w*/*w* HNO_3_. Collected samples were properly diluted with 2% *w*/*w* HNO_3_ before each measurement, down to Ag^+^ concentrations in the range 1–100 ppb. Untreated samples were used as blank controls, and absence of ionic release from glass vials was also tested, using the same experimental procedures.

### 3.4. Assessment of Entire Nanoparticle Release

Pristine and modified leather and leatherette samples were cut into 3.5-cm-side squares. All samples were then covered (on the Ag-treated side) with a 2-mL quantity of ultrapure water (Milli-Q, 18.2 MΩ), having an approximate surface of 10 cm^2^. Release of entire NPs was evaluated after 4 h. After this time, small aliquots of the liquid were sampled and deposited on TEM grids (Formvar^®^/Cu 300 mesh, Assing S.pA., Rome, Italy) for subsequent morphological characterization on a Fei Tecnai 12, operated at 120 kV with a LaB_6_ filament. The remaining fraction of each sample was used for SP-ICP-MS characterizations. The latter were performed in collaboration with Perkin-Elmer, using a NexION-350-X, managed with a proper software application developed and provided by the producer (Syngistix™ Nano Application Module, PerkinElmer, Inc., Waltham, MA, USA). Main experimental parameters are listed in Table 5.

A control sample containing AgNPs with known size and concentration, free of significant amounts of metal ions, was used to calibrate the spectrometer in this operation mode. In fact, this step is crucial in distinguishing between signal contribution due to AgNPs, and the background of silver ions [34]. Reference AgNPs were synthesized according to the procedure proposed by Ales Panáček [35], which provides size-controlled particles (≈60 nm) through the reduction of [Ag(NH_3_)_2_]^+^ complex formed from the mixing of AgNO_3_ and NH_3_ in equal concentrations. AgNP reference samples were characterized by UV-Vis spectroscopy and TEM microscopy (refer to Figure S1), to confirm the presence of AgNPs having the expected size.

### 3.5. Biological Characterizations before and after Abrasion

The biological characterizations were performed before and after Taber tests, aiming at evaluating the durability of the antibacterial capability of the silver treated samples and their biocompatibility also after an abrasion of the materials surface. The Taber test was performed on natural leather and leatherette according to Standard ASTM D7255, by using CS-10 wheels for 2000 cycles and a load of 500 g.

#### 3.5.1. Antibacterial Tests

For microbiological characterization, Ag-treated samples were prepared by using a silver solution containing a significantly lower percentage of silver precursor, namely 0.1% *w*/*w*. The antibacterial activity of pristine and modified leather and leatherette specimens was tested on *E. coli* (ATCC 25922, inoculated bacterial density 2.7 × 10^8^ CFU/mL) and *S. aureus* (ATCC 29213, inoculated bacterial density 3 × 10^7^ CFU/mL) through Agar diffusion tests, according to Standard ‘SNV 195920-1992’. An aliquot of 100 μL of the bacterial suspension was plated on nutrient Agar; then, samples were placed over bacteria and incubated in an oven at 37 °C, overnight. After incubation, bacterial growth inhibition area was evaluated and labelled according to antibacterial capability levels. If the sample is completely covered by bacteria, the Standard defines the antibacterial capability as “insufficient’; if the inhibition area to bacterial growth around each sample is larger than 1 mm, the antibacterial efficacy is labelled as “good” [36]. The antibacterial efficacy (ABE %) of pristine and modified leather and leatherette was also examined through bacterial enumeration tests performed according to the serial dilution method. The pristine and silver treated samples (0.5 g) were introduced in 6 mL of nutrient broth inoculated with *S. aureus* and *E. coli* (1.5 × 10^5^ colony forming units CFU/mL) and incubated at 37 °C in a shaking incubator for 12 h. Then, serial dilutions were performed and 100 μL of each dilution were spread plated on agar plates and incubated at 37 °C for 24 h. The bacterial colonies (CFU) grown on the agar plates after incubation were counted and the antibacterial efficacy (ABE %) of the samples was calculated according to the following Equation (1) [37]:

ABE (%) = (*V_c_* − *V_t_*)/ *V_c_* × 100 (1)

where *V_c_* and *V_t_* are the numbers of viable bacterial colonies grown in the presence of untreated and treated sample respectively.

#### 3.5.2. MTT Assay

Murine fibroblasts 3T3 were maintained in Dulbecco’s Modified Eagle Medium (DMEM, Sigma Aldrich, Milan, Italy) supplemented with 10% fetal bovine serum (FBS), antibiotics (100 mg/mL streptomycin and 100 U/mL penicillin) and 2 mM l-glutamine. Cultures were supplied with fresh medium every 3 days and incubated at 37 °C in a humidified 5% CO_2_ atmosphere (Heracell, Thermo Scientific, Waltham, Massachusetts, US). The biocompatibility of the silver treated samples was evaluated through MTT assay (Sigma Aldrich, Milan, Italy) and an indirect method, aiming at assessing the safety of the materials and relating the results to those obtained by the investigations on the silver release. For this purpose, both the concentrations of silver precursor, namely 0.1 and 4% *w*/*w*, were tested before and after Taber abrasion tests. The extraction method consisted of incubating the samples in 6 mL of PBS solution and in collecting their extracts after 24 h for MTT assays. After incubation, the indirect contact tests were performed in triplicate after 1, 3, 7 days of culture. The samples were let in contact with 6 mL of PBS solution and their extracts were collected after 24 h for MTT assays. The eluates were placed in contact with cell cultures and indirect contact tests were performed in triplicate after 1, 3, 7 days of culture. MTT solution (5 mg/mL in PBS, Sigma Aldrich) was added to reach a final concentration of 0.5 mg/mL and the plates were incubated at 37 °C for 2 h. After the assay, the blue formazan reaction product was dissolved by adding 1 mL of isopropanol to each well. Then, the supernatants were centrifuged at 13,000× *g* for 5 min and the solubilized formazan was measured at 550 nm using a spectrophotometer (V-1200, VWR International, Radnor, Pennsylvania, US).

## 4. Conclusions

A photo-assisted protocol for AgNP deposition was used to modify natural and synthetic leathers. SEM and XPS analyses demonstrated that the process was effective for the deposition of AgNPs onto both substrates, without altering their surface chemical composition significantly. XPS evidences suggested that conversion of silver precursor into nanostructured Ag clusters was almost complete: no signals related to AgNO_3_ were detectable. ICP-MS measurements demonstrated that modified specimens were able to provide a modulated release of antibacterial ions in physiological solution, up to 24 h. The absence of entire NPs release was demonstrated by SP-ICP-MS; such results were validated using TEM analysis on the same contact solution. Ruling out the existence of a significant AgNP release from treated materials, the occurrence of toxicological risks could be excluded a priori. The safety of the materials was assessed through biocompatibility tests, which demonstrated that no adverse effect on human health could be associated with release of silver from the coatings. On the other hand, microbiological tests on model *E. coli* and *S. aureus* showed that the proposed nano-functionalized materials exerted a marked and durable inhibitory effect.

## Figures and Tables

**Table 1:** Surface elemental composition estimated by X-ray photoelectron spectroscopy (XPS) of leather samples treated with 4% *w*/*w* Ag precursor. Error is expressed as the larger value between the error associated to a single quantification (0.2% for silver, 0.5% for other elements) and one standard deviation, calculated on at least three replicate analyses. Data on pristine samples are reported for comparison.

Sample	Pristine	Treated
Element	At%	At%
C	69 ± 4.0	58 ± 3.0
N	0.5 ± 0.5	0.9 ± 0.5
O	21 ± 3.0	23.6 ± 0.6
Si	9 ± 1.0	17 ± 3.0
Ag	/	0.5 ± 0.2
Br	0.5 ± 0.5	≤0.5

**Table 2:** Leather C1s signal components. Error is expressed as the larger value between the error associated to a single quantification and one standard deviation, calculated on at least three replicate analyses.

Sample		Pristine	Treated
Position	Attribution	Relative %	Relative %
284.8 ± 0.1	C–C	60 ± 2	57 ± 4
286.2 ± 0.2	C–O, C–N	29 ± 3	33 ± 1
287.4 ± 0.3	C=O	8.5 ± 0.9	5.7± 0.6
289.1 ± 0.3	HN–COO	2.5 ± 0.6	4.3 ± 0.7

**Table 3:** Surface elemental composition estimated by XPS of leatherette samples treated with 4% *w*/*w* Ag precursor. Error is expressed as the larger value between the error associated to a single quantification (0.2% for silver, 0.5% for other elements) and one standard deviation, calculated on at least three replicate analyses. Data on pristine samples are reported for comparison.

Sample	Pristine	Treated
Element	At%	At%
C	68 ± 2	62.3 ± 0.9
O	21 ± 1	25.1 ± 0.5
N	0.8 ± 0.5	1.3 ± 0.5
Si	10.2 ± 0.5	10.7 ± 0.6
Ag	/	0.6 ± 0.2

**Table 4:** Leatherette C1s signal components. Error is expressed as the larger value between the error associated with a single quantification and one standard deviation, calculated on at least three replicate analyses.

Sample		Pristine	Treated
Position	Attribution	Relative %	Relative %
284.8 ± 0.1	C–C	77 ± 2	81 ± 3
286.5 ± 0.2	C–O, C–N	20 ± 2	15 ± 2
289.1 ± 0.2	HN–C=O	3 ± 1	4 ± 2

**Table 5:** Single particle inductively coupled plasma-mass spectrometry (SP-ICP-MS) instrumental parameters.

**Dwell time (μs)**	50
**Settling time (μs)**	0
**Scan time (s)**	60
**Sample flow rate (mL/min)**	0.276

## Supplementary Materials

The following are available online at http://www.mdpi.com/2079-4991/7/8/203/s1, Figure S1: UV-Vis spectrum (a) and TEM micrographs (b,c) of control AgNPs synthetized by the chemical route described in the experimental section; Table S1: Operating conditions used for ICP-MS analysis; Figure S2: Representative pictures obtained by bacterial enumeration tests on *E. coli* before (a–d) and after (A–D) surface abrasion on pristine (UT) and Ag-treated (T) leather and leatherette; Figure S3: Bacterial enumeration tests on *S. aureus* before (e–h) and after (E–H) surface abrasion on pristine (UT) and Ag-treated (T) leather and leatherette.
