# Supplementary material for: Spectroscopic Characterization and Nanosafety of Ag-Modified Antibacterial Leather and Leatherette
Source: Nanomaterials (Basel). 2017 Jul 29;7(8):203. doi: 10.3390/nano7080203 (PMC5575685; doi:10.3390/nano7080203)
Supplement: Supplementary file 1 [file nanomaterials-07-00203-s001.pdf]

Nanomaterials

Electronic Supplementary Material

Spectroscopic characterization and nanosafety of Ag-modified antibacterial leather and leatherette

Maria Chiara Sportelli, Rosaria Anna Picca, Federica Paladini, Annarosa Mangone, Lorena Carla Giannossa, Cinzia Di Franco, Anna Lucia Gallo, Antonio Valentini, Alessandro Sannino, Mauro Pollini, Nicola Cioffi

Table S1. Operating conditions used for ICP-MS analysis. [a] atomic mass unit.

**Plasma Power**	1600 W
**Plasma (Ar) gas flow**	18 mL/min
**Auxiliary(Ar) gas flow**	1.2 mL/min
**Sample transport (Ar)**	1.0 mL/min
**Collision chamber (He)**	2.0 mL/min
**Nebulizer, spray chamber and flow**	Meinhard, Cyclonic, 1 mL/min
**Isotopes monitored**	^{107}Ag, ^{109}Ag
**Dwell time per AMU[a]**	50 ms
**Integration time**	1 s

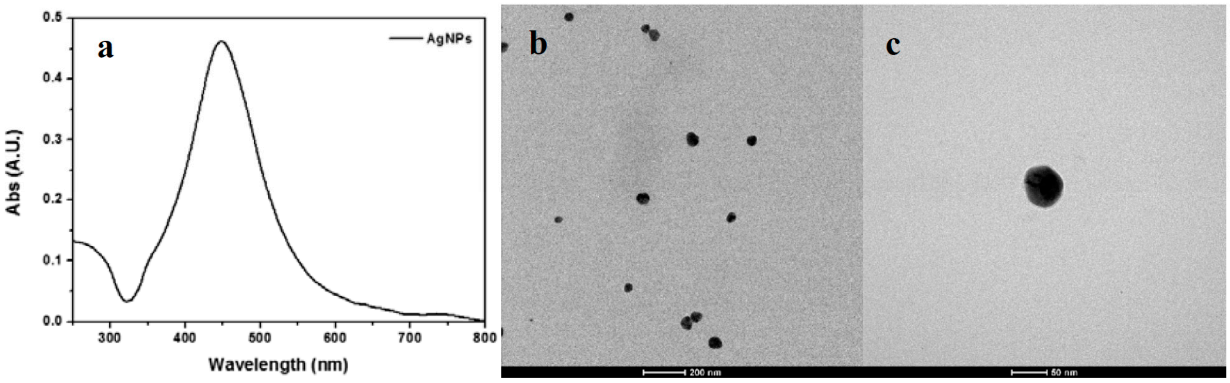

Figure S1: UV-Vis spectrum (a) and TEM micrographs (b, c) of control AgNPs synthetized by the chemical route described in the experimental section.

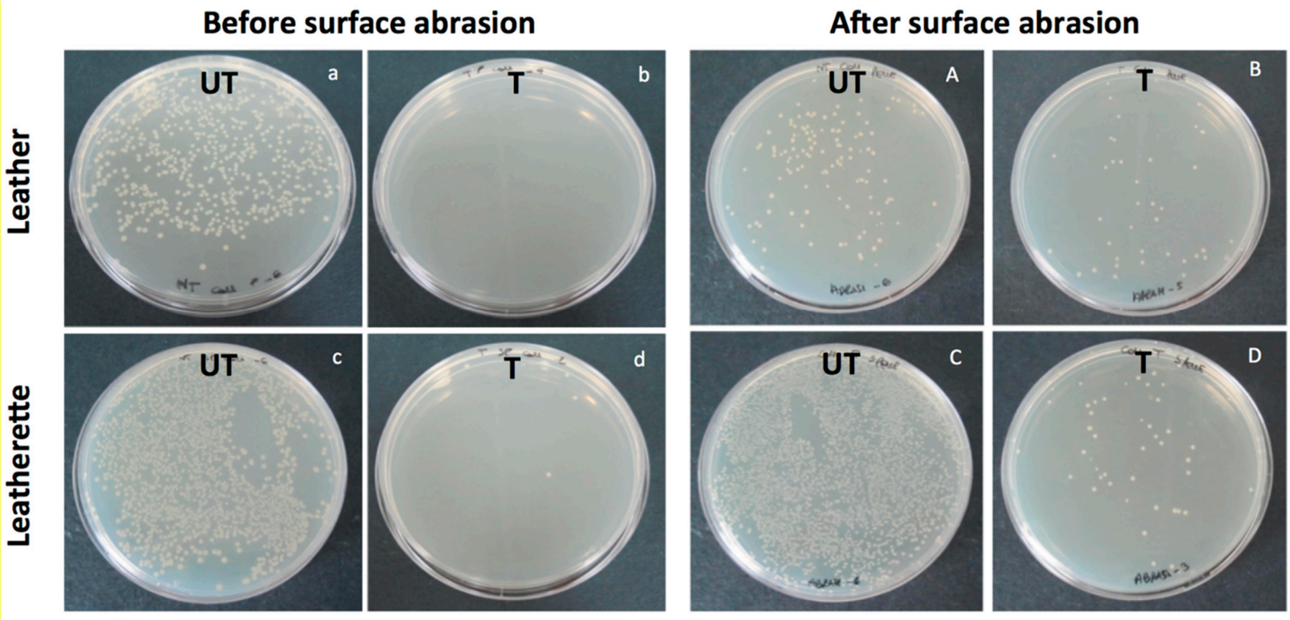

Figure S2: Agar diffusion tests on *E. coli* before (a-d) and after (A-D) surface abrasion on pristine (UT) and Ag-treated (T) leather and leatherette.

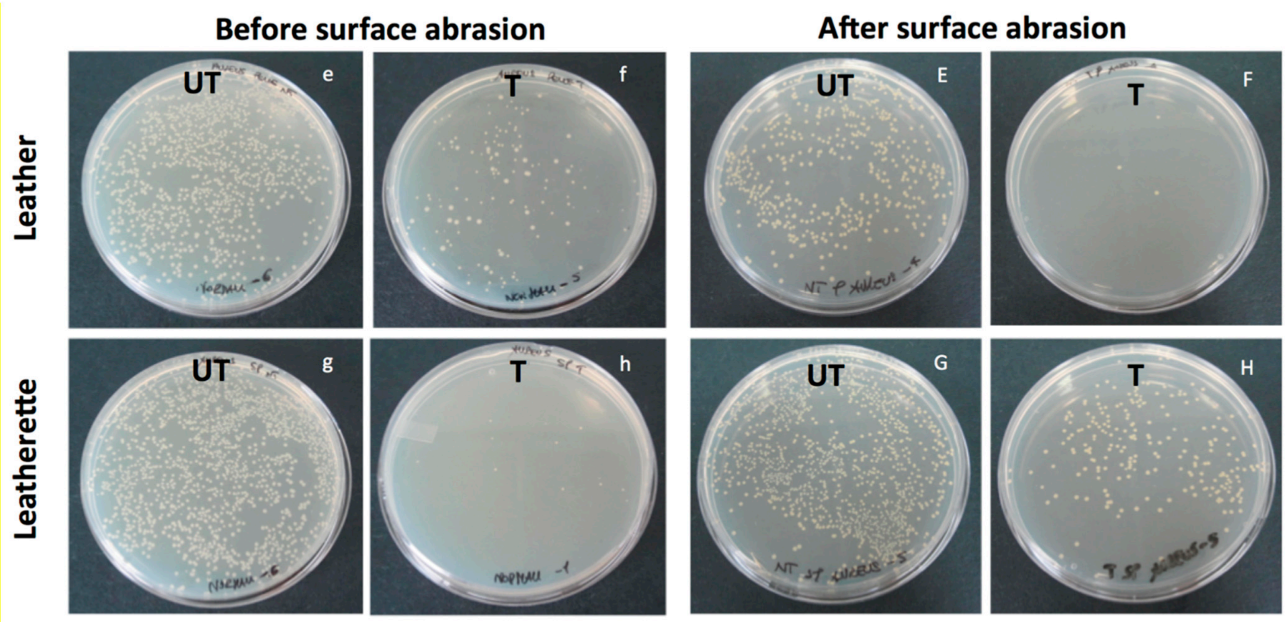

Figure S3: Agar diffusion tests on *S. aureus* before (e-h) and after (E-H) surface abrasion on pristine (UT) and Ag-treated (T) leather and leatherette.
